# LncRNA XIST promotes bladder cancer progression by modulating miR-129-5p/TNFSF10 axis

**DOI:** 10.1007/s12672-024-00910-8

**Published:** 2024-03-06

**Authors:** Yu-Lin Kong, Hui-Dan Wang, Meng Gao, Sheng-Zhong Rong, Xiao-Xia Li

**Affiliations:** Department of Epidemiology, School of Public Health, Mudanjiang Medical University, 3 Tong Xiang Street, Mudanjiang, 157011 Heilongjiang China

## Abstract

**Background:**

The differential expression, biological function, and ceRNA regulatory mechanism of lncRNA XIST in bladder cancer (BC) were investigated, and its clinical values for the early diagnosis of bladder cancer patients were elucidated.

**Methods:**

qRT-PCR was employed to detect the expression patterns of lncRNA XIST, miR-129-5p and TNFSF10. The biological functions were measured by CCK8 assay, wound healing assay and transwell assay. Bioinformatics analysis and Dual-Luciferase reporter assay were employed to evaluate the interactions between the lncRNA XIST, miR-129-5p and TNFSF10.

**Results:**

LncRNA XIST and TNFSF10 were highly expressed and miR-129-5p was low expressed (*P* < 0.05) in bladder cancer cell line. The depletion of lncRNA XIST inhibited BC proliferation, migration and invasion. Mechanistically, lncRNA XIST could sponge miR-129-5p to regulate TNFSF10 expression in bladder cancer. Furthermore, compared with adjacent tissues, lncRNA XIST and miR-129-5p were lowly expressed (*P* < 0.01) in bladder cancer tissues, and TNFSF10 was highly expressed (*P* < 0.001). miR-129-5p and TNFSF10 were associated with the risk of bladder cancer (*P* < 0.05); the difference in AUC values for the diagnosis of bladder cancer by lncRNA XIST (AUC = 0.739), miR-129-5p (AUC = 0.850) and TNFSF10 (AUC = 0.753) was statistically significant (*P* < 0.01), and the three genes combined AUC was 0.900, 95%CI was 0.842–0.958 with a sensitivity of 83.3% and specificity of 86.7%.

**Conclusion:**

XIST, an elevated lncRNA in bladder cancer, inhibition of which could suppress the progression of BC. LncRNA XIST and miR-129-5p could form ceRNA to regulate the expression of TNFSF10.

## Introduction

Bladder cancer (BC) is one of the most common malignancies in the urinary system. The incidence of BC has shown an upward trend in recent years which ranks tenth worldwide. Its global incidence ranks sixth and mortality ranks ninth in male malignant tumors [1]. Currently, BC is mainly treated by surgery, supplemented by chemotherapy and radiotherapy, however, its recurrence and metastasis rates remain high and the overall survival rate has not improved significantly [2]. Thus, an in-depth exploration of the pathogenesis, recurrence, and metastasis mechanisms of BC and the exploration of effective biomarkers and potential therapeutic targets are crucial for the prevention and treatment of BC.

Accumulating evidence has indicated that long non-coding RNAs (lncRNAs) are aberrantly expressed in different malignancies and are able to regulate tumor cell growth and development through multiple signaling pathways, including tumor cell proliferation, migration, invasion, recurrence and metastasis [3, 4]. LncRNAs are rich in microRNA(miRNA) binding sites and serve as competing endogenous RNAs (ceRNAs) and miRNA sponges, thus reducing their interference with downstream genes and facilitating downstream mRNA translation [5].

Long non-coding RNA X chromosome inactive specific transcript (lncRNA XIST), the gene product of XIST, is a major regulator of transcriptional silencing of the mammalian X chromosome and its encoding gene is located at human chromosome Xq13.2 [6], has been demonstrated to be upregulated in most tumors and to promote tumorigenesis, progression, and metastasis [7–10]. Hu et al. found that lncRNA XIST level was high in BC and promoted bladder cancer cell proliferation and migration [11–13], few studies have been reported on regulating the lncRNA XIST/miR129-5p/TNFSF10 axis.

The study screened differentially expressed lncRNAs in BC focusing on elucidating how lncRNA XIST affected the pathogenesis of BC and further identifying its diagnostic efficacy. Our study illuminated that lncRNA XIST mediates the miR129-5p/TNFSF10 axis, thereby aggravating BC progression and aimed to provide new perspectives for the trearment of BC.

## Materials and methods

### BC patients and tissue specimens

From 2019 to 2021, sixty pairs of tumor tissues and adjacent tissues were totally collected from patients with bladder cancer at Harbin Medical University Cancer Hospital (Harbin, China). All specimens were pathologically confirmed as bladder cancer and did not receive preoperative radiotherapy or chemotherapy. Histological diagnosis of all cases was evaluated according to criteria established by the World Health Organization (WHO). All samples were immediately frozen in -80 °C after surgical resection. Written informed consent was obtained from each participant and with approval from the ethics committee of Mudanjiang Medical University (No.2018-MYGZR01).

### RNA-Seq and bioinformatics analysis

RNA-seq of T24 and SV-HUC-1 cell lines were performed using an Illumina HiSeq™ 2000 (Illumina, USA). Quality control was performed using Fast QC and clean reads were aligned to the human genome(hg19) using Tophat. Differentially expressed lncRNA were identified by DEseq2, with *P* value < 0.05 and |log2(foldchange)|> 1 as the thresholds to evaluate the statistical significance of the lncRNA expression differences.

Three bioinformatics databases including miRDB (http://mirdb.org/), miRcode (http://www.mircode.org/) and Starbase (https://starbase.sysu.edu.cn/) were employed to predict the potential bindings of miRNAs to individual lncRNA. In addition, three algorithms including miRanda, RNA22 and microT were adopted to analyze possible target mRNAs for miRNAs. The binding sites were predicted using Starbase.

### Cell culture

BC cell line T24, human normal uroepithelial cell line SV-HUC-1 and human renal epithelial cell line 293 T were purchased from China Center for Type Culture Collection (WuHan, China), and were allowed to stand in McCoy'5A medium (Termo Fisher Scientifc, 16600082), Roswell Park Memorial Institute 1640 medium (RPMI-1640; Termo Fisher Scientifc, 11875119) and Dulbecco modifed Eagle Medium (DMEM; Termo Fisher Scientifc, 11966025), respectively. All media were supplemented with 10% fetal bovine serum (FBS; VivaCell, Shanghai, China, C04001) and 1% penicillin—streptomycin at 37 °C with a 5% CO_2_ atmosphere.

### RNA extraction and quantitative reverse transcription PCR (qRT-PCR)

Total RNA from cells and tissues were extracted using Trizol reagent (Invitrogen, USA) following the manufacturer’s instructions. For lncRNAs and mRNAs, Transcriptor First Strand cDNA synthesis Kit (Roche, 0489703000) was applied for reverse transcriptions. qRT-PCR was performed using FastStart Universal SYBR Green Master (Rox) (Roche, 4913850001) on the instrument of ABI 7500 (Thermo, USA). For miRNAs, SanPrep Column miRNA Extraction Kit(Sangon Biotech, B518811), miRNA First Strand cDNA Synthesis (Sangon Biotech, B532453) and miRNA qPCR Kit (Sangon Biotech, B532461) were adopted. Relative gene expressions were determined using the 2^–ΔΔCT^ method. The primers were described in Table 1.

**Table 1:** Primer sequences used for qRT-PCR

Gene	Primer	Sequence (5′-3′)
GADPH	Forward	GCATCCTGGGCTACACTG
	Reverse	TGGTCGTTGAGGGCAAT
U6	Forward	CGCTTCGGCAGCACATATACTAAAATTGGAAC
	Reverse	GCTTCACGAATTTGCGTGTCATCCTTGC
	RT Primer	AAAATATGGAACGCTTCACG
lncRNA XIST	Forward	AGCAGGTCAGGCAGAGGAAGTC
	Reverse	CCCGATACAACAATCACGCAAAGC
TNFSF10	Forward	TTACCAACGAGCTGAAGCAGATGC
	Reverse	GCTGACGGAGTTGCCACTTGAC
miR-129-5p	Forward	TGCGCTTTTTGCGGTCTGG
	Reverse	AGTGCAGGGTCCGAGGTATT
	RT Primer	GTCGTATCCAGTGCAGGGTCCGAGGTATTCGCACTGGATACGACGCAAGC

### Cell transfection

When cell confluence reached 60%, cell transfection was performed using RNA TransMate (Sangon Biotech, E607402). The siRNAs were synthesized by Sangon (Shanghai, China) and sequences were listed as Table 2.

**Table 2:** Primer sequences used for cell transfection

Name		Sequence (5′-3′)
siNC	Sense	UUCUCCGAACGUGUCACGUTT
	Anti-sense	ACGUGACACGUUCGGAGAATT
siXIST-1	Sense	GUACAAGGGACUAGUUAAATT
	Anti-sense	UUUAACUAGUCCCUUGUACTT
siXIST-2	Sense	GGACAUUAUUGGACAUUAATT
	Anti-sense	UUAAUGUCCAAUAAUGUCCTT
siXIST-3	Sense	GGAAUGAACUAUUGGCUUATT
	Anti-sense	UAAGCCAAUAGUUCAUUCCTT

### Cell proliferation assay

Cell proliferation was examined using Cell Counting Kit-8 (CCK-8; BioSharp, China) based on the the provided guidelines. Briefly, transfected cells (5 × 10^3^ cells/well) were seeded into 96 well plates, and 10 μl of CCK-8 was added to each well. The optical density (OD) was read at 450 nm at different time points (24, 48, 72 h).

### Cell migration and invasion assay

Stably transfected cells were inoculated into six well plates (5 × 10^5^ cells/well). When 90% confluence was reached, a wound was created by a 10 µl pipette tip. The movement of cells was captured by a phase-contrast microscope at different time points (0,6,12 h). A total of 200 μl cells suspension (1 × 10^5^ cells) filled with serum-free medium was added into the upper chamber with polycarbonate membrane coated with Matrigel in advance, while the bottom chamber was filled with 600 μl medium with 20% FBS. After incubating for 24 h, the invasive cells on the outer membrane were immobilized by paraformaldehyde and stained with crystal violet solution, and five different visual fields were randomly selected to be photographed under a microscope.

### Luciferase reporter assay

Dual-Luciferase reporter vector with the wild-type (WT) or mutated (MUT) versions of lncRNA XIST and TNFSF10 that containing binding sites or mutant sites with miR-129-5p were constructed based on the pmiR-GLO luciferase Vector (Promega, USA). The miR-129-5p or NC mimics and vectors were co-transfected into 293 T cells for 48 h. Then the Dual-Luciferase Reporter Assay System (Promega, USA) was utilized to evaluate the luciferase activity of each group according to the provided protocol, with Renilla Luciferase activity being used as a normalization control.

### Statistical analysis

SPSS 25.0 and GraphPad Prism 8.0 were utilized for statistical analysis, with *P* < 0.05 as the significance threshold. Statistical comparisons were performed using Student’s t-tests, Wilcoxon signed-rank tests or Pearson chi-squared tests as appropriate. The diagnostic indexes of each biomarkers were performed using receiver operating characteristic (ROC) curves, and the optimal cut-point value was determined according to the Youden index. To evaluate the variance of various biomarker combination, binary logistic regression and ROC curves were adopted.

## Results

### Identification of deregulated lncRNAs in BC Cells

To explore the expression profiles of genome-wide lncRNA in BC, T24 and SV-HUC-1 cell lines were used for RNA-seq. We identified 3571 differentially expressed lncRNAs (870 up-regulated and 856 down-regulated) using log_2_FC (fold change) ≥ 1 and adjusted *P* value < 0.05 as threshold parameters, as the top ten lncRNAs showed in Table 3. LncRNA XIST was selected for this study mainly because it was highly differentially expressed and less studied in BC.

**Table 3:** Differentially expressed lncRNAs in BC

lncRNA	Log_2_(FC)	*P* value
CCAT1	12.73379092	1.34E-26
XIST	11.48862747	3.58E-210
FAM225B	10.60531477	1.28E-18
LBX1-AS1	9.740805023	1.61E-15
PICSAR	9.410366566	2.44E-27
FAM230E	9.145501376	3.67E-14
PWARSN	8.216856319	6.55E-11
FAM230F	8.043261146	5.43E-11
KCNMB2-AS1	7.788760558	1.08E-09
PWAR5	7.627372859	2.74E-09

### LncRNA XIST/ miR-129-5p/ TNFSF10 axis was conducted

**Fig. 1:**
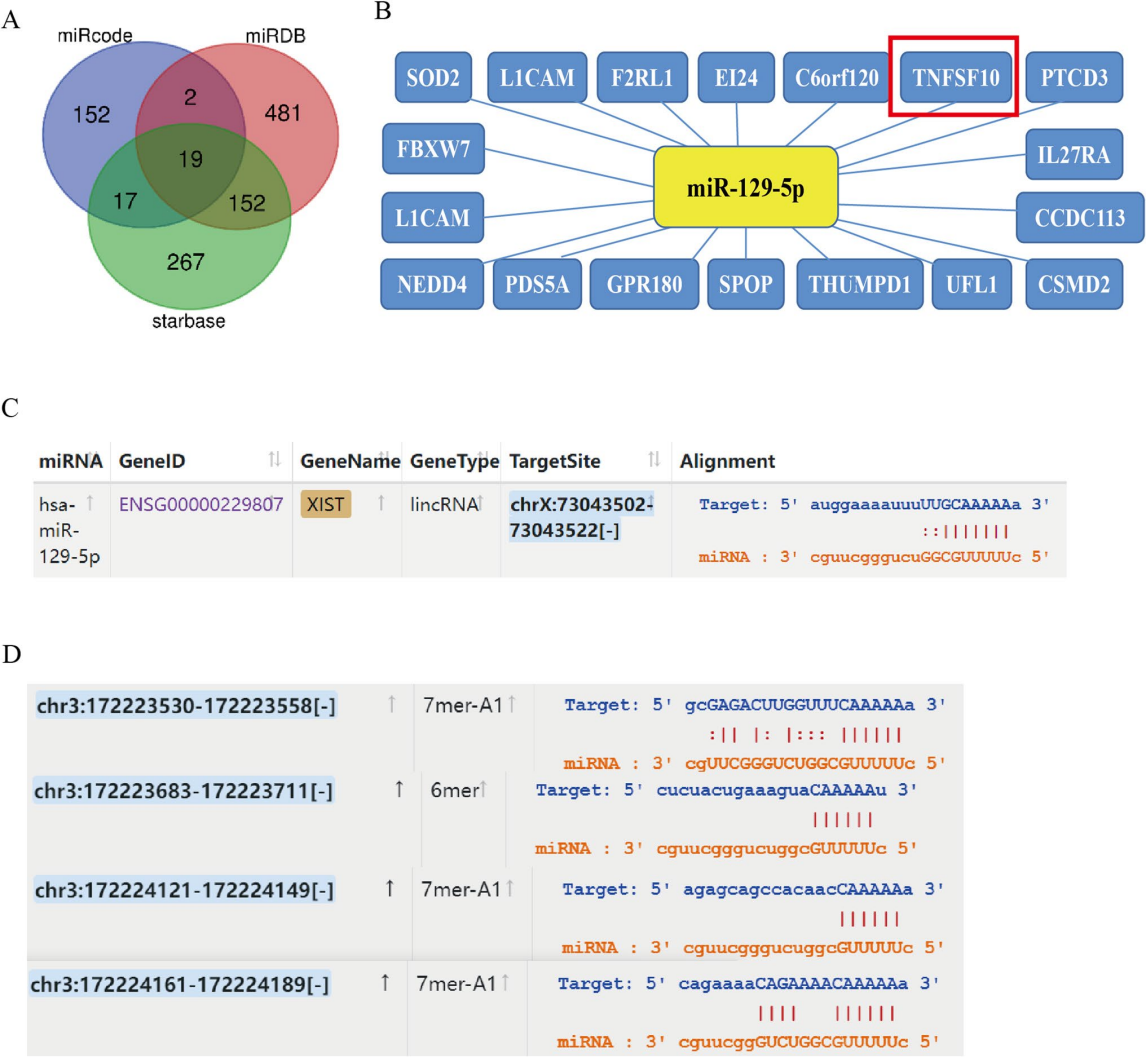
Screening of targeting miRNAs and mRNAs and prediction of binding sites. **A** The three bioinformatics tools showed an overlap of 19 miRNAs which targeted lncRNA XIST using a Venn diagram. **B** The target mRNAs of miR-129-5p were screened out by bioinformatics algorithm. **C** The putative binding site between lncRNA XIST and miR-129-5p was predicted by StarBase. **D** The putative binding site between miR-129-5p and TNFSF10 was predicted by StarBase

Previous studies have indicated that lncRNAs function as miRNAs sponges to regulate biological events, and we predicted the target miRNAs of lncRNA XIST and target mRNAs of the chosen miRNA by bioinformatics algorithm. Among these target miRNAs, we noted miR-129-5p as a potential target while a total of 19 overlapping miRNAs were screened (Fig. 1A). And a total of 18 target genes binding to miR-129-5p were screened (Fig. 1B). Among them, TNFSF10 was found to be highly differentially expressed in RNA-seq and was included. To confirm the miR-129-5p as the sponging target of lncRNA XIST, we first calculated the number of potential binding sites between lncRNA XIST conserved sequences and miR-129-5p by StarBase (Fig. 1C). Meanwhile, we also calculated the number of potential interaction sites between miR-129-5p and TNFSF10 (Fig. 1D). In summary, the lncRNA XIST/ miR-129-5p/ TNFSF10 axis provided a theoretical basis for its regulatory role in BC and provided a theoretical basis for subsequent experimental validation.

### Levels of lncRNA XIST, miR-129-5p and TNFSF10 expression in BC cell lines

**Fig. 2:**
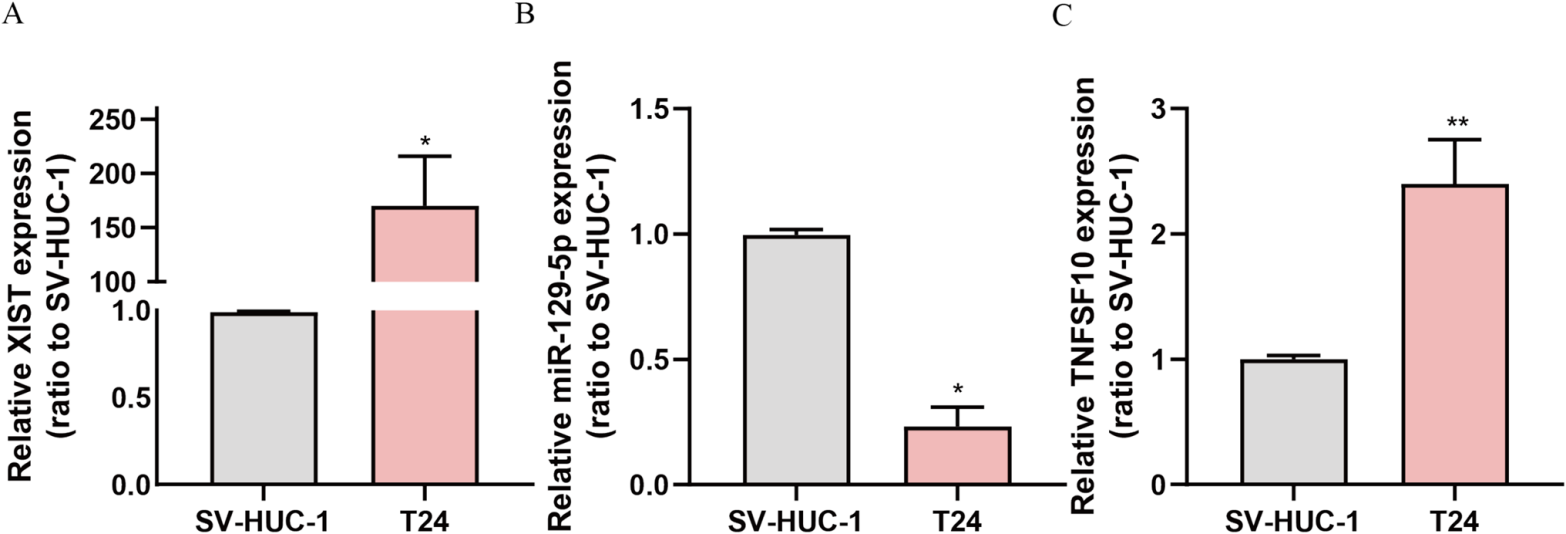
Levels of lncRNA XIST, miR-129-5p and TNFSF10 expression in BC. **A**–**C** qRT-PCR was used to detect the expression levels of lncRNA XIST, miR-129-5p and TNFSF10 in BC cell line T24 and normal uroepithelial cell line SV-HUC-1, demonstrating that lncRNA XIST and TNFSF10 were upregulated and miR-129-5p was downregulated. ^*^ *P* < 0.05; ^**^ *P* < 0.01

We explored the expression of lncRNA XIST in BC cell line (T24) and normal uroepithelial cell line (SV-HUC-1) and found that the expression of lncRNA XIST and TNFSF10 was considerably increased in the T24 cells, in comparison with SV-HUC-1 cells (Fig. 2A, C). In addition, the relative expression of miR-129-5p was lower than that of the control group (Fig. 2B).

### Silence of lncRNA XIST suppressed cell proliferation, migration and invasion in BC

**Fig. 3:**
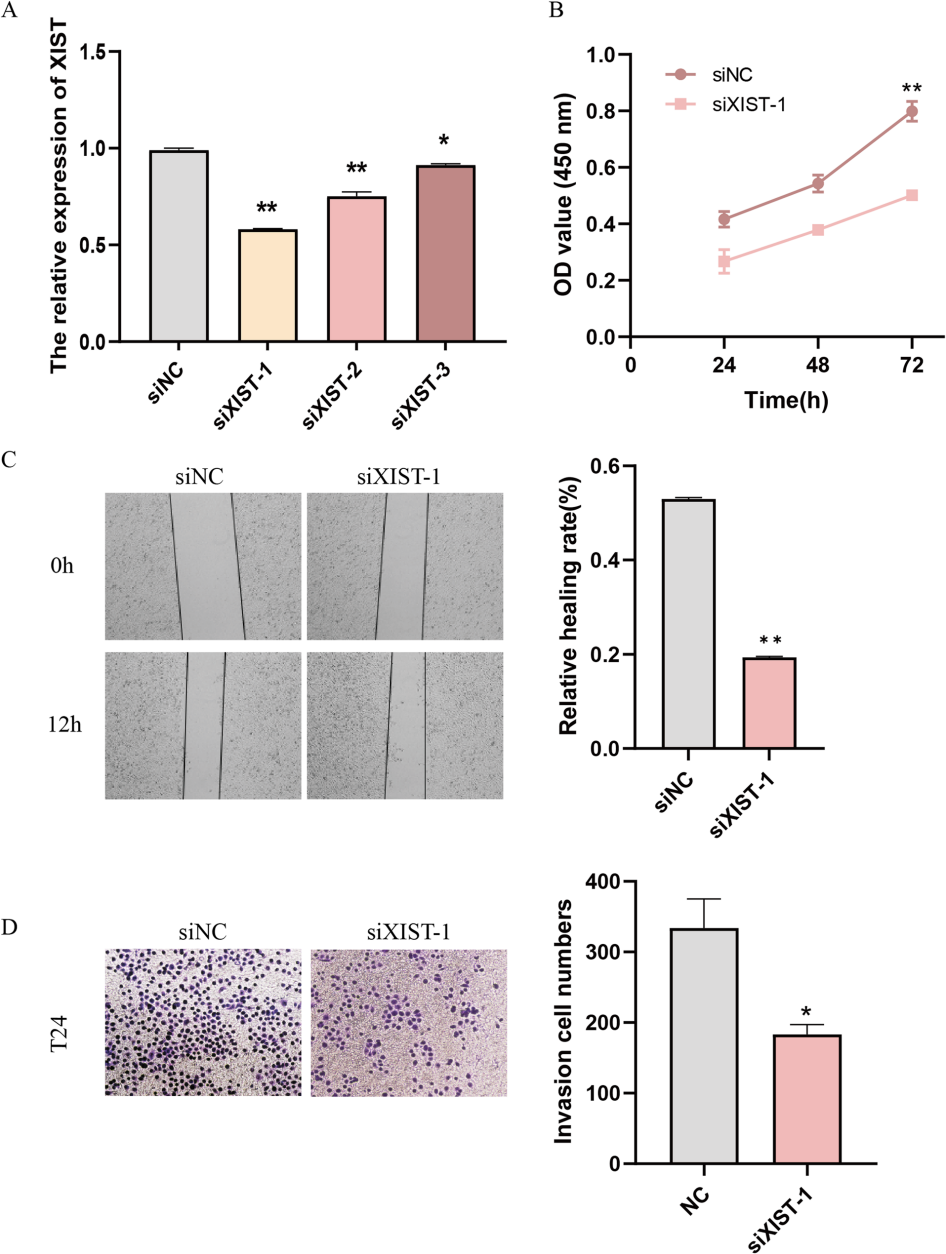
Silence of lncRNA XIST suppressed cell proliferation, migration and invasion in BC. **A** Transfection efficiency of siXIST was confirmed by qRT-PCR. **B** CCK-8 assay was used to test the cell proliferation. **C** Wound-healing assay was used to test the cell migration. **D** Transwell assay was used to test the cell invasion. ^*^ *P* < 0.05; ^**^ *P* < 0.01

Given that lncRNA XIST upregulation is associated with BC, we next purchased siRNAs targeting lncRNA XIST (siXIST-1, siXIST-2 and siXIST-3) and transfected them into T24 cells to identify the biological function of lncRNA XIST in BC. qRT-PCR was performed to confirm the siRNAs were both effective (Fig. 3A). Concomitantly, the proliferation, migration and invasion of T24 were dramatically abrogated compared to control cells (Fig. 3B, C and D). These evidences indicated that lncRNA XIST might contribute to the malignant phenotypes of BC cells in vitro.

### LncRNA XIST/miR-129-5p/TNFSF10 axis in BC

**Fig. 4:**
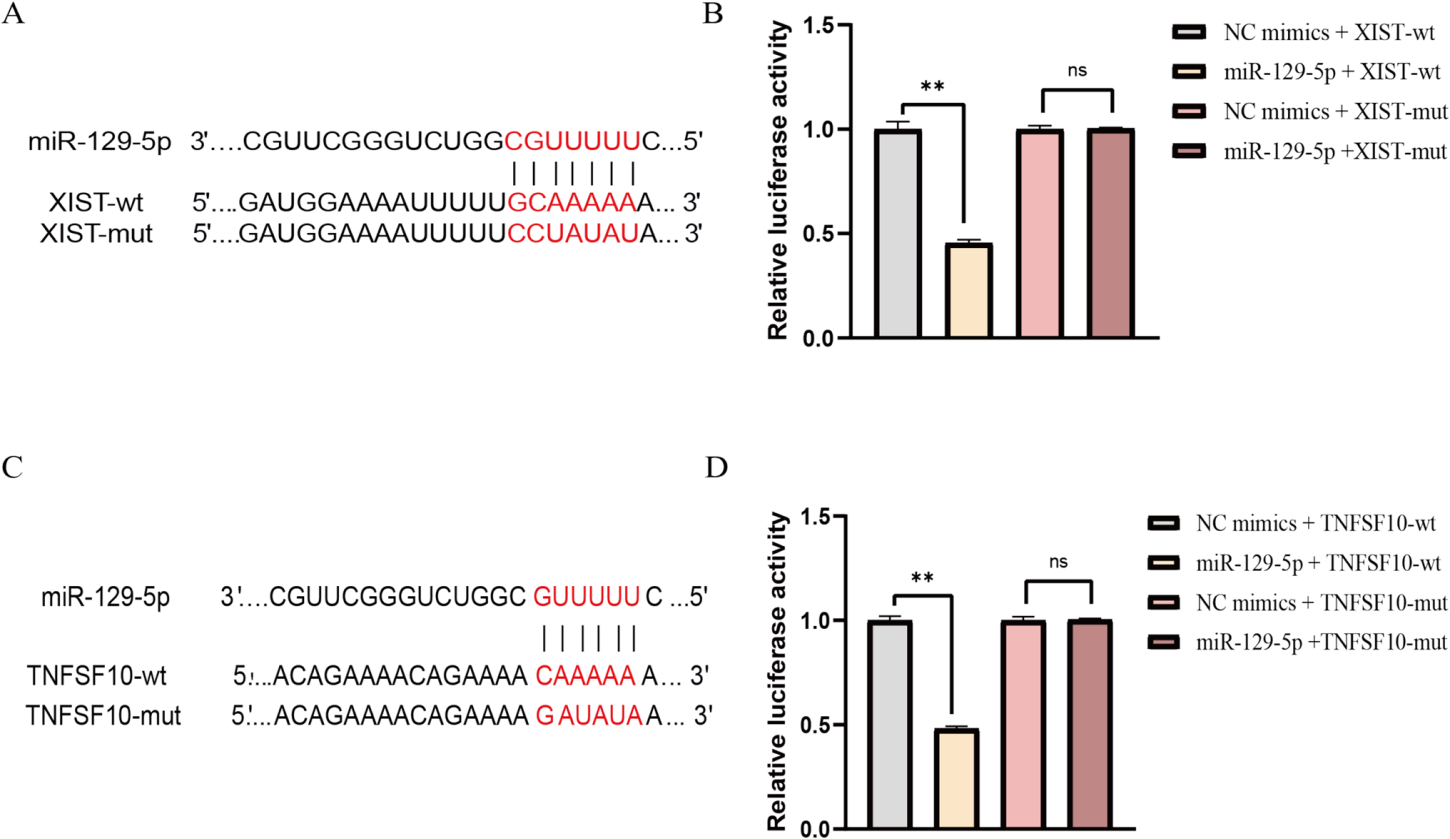
LncRNA XIST/miR-129-5p/TNFSF10 axis in BC. **A** The putative binding site between lncRNA XIST and miR-129-5p was exhibited. **B** Luciferase activity was examined cotransfected with h-XIST-3UTR-WT or h-XIST-3UTR-MUT and miR-NC or miR-129-5p mimics. **C** The putative binding site between miR-129-5p and TNFSF10 was exhibited. **D** Luciferase activity was examined cotransfected with h-TNFSF10-3UTR-WT or h-TNFSF10-3UTR-MUT plasmids and miR-NC or miR-129-5p mimics

To confirm these bioinformatic data, we designed binding sites (Fig. 4A, C). To verify the prediction of lncRNA XIST, luciferase reporter assay was conducted by co-transfection of miR-129-5p mimics and miR-NC with luciferase reporter into WT and MUT, respectively. Compared to the miR-NC and h-XIST-3UTR-WT cotransfected groups, the luciferase reporter activity of miR-129-5p mimics and h-XIST-3UTR-WT cotransfected groups was significantly lower (Fig. 4B). The luciferase reporter activity of miR-NC and h-XIST-3UTR-MUT cotransfected group had no difference with miR-129-5p mimics and h-XIST-3UTR-MUT cotransfected group (Fig. 4B). Besides, as verified by assays, h-TNFSF10-3UTR-WT greatly hindered the luciferase activity exhibited by miR-129-5p instead of the mutant form (Fig. 4D). All those results further demonstrated that lncRNA XIST could sponge miR-129-5p to regulate TNFSF10 expression in bladder cancer through these binding sites.

### Levels of lncRNA XIST, miR-129-5p and TNFSF10 expression in BC tissues

**Fig. 5:**
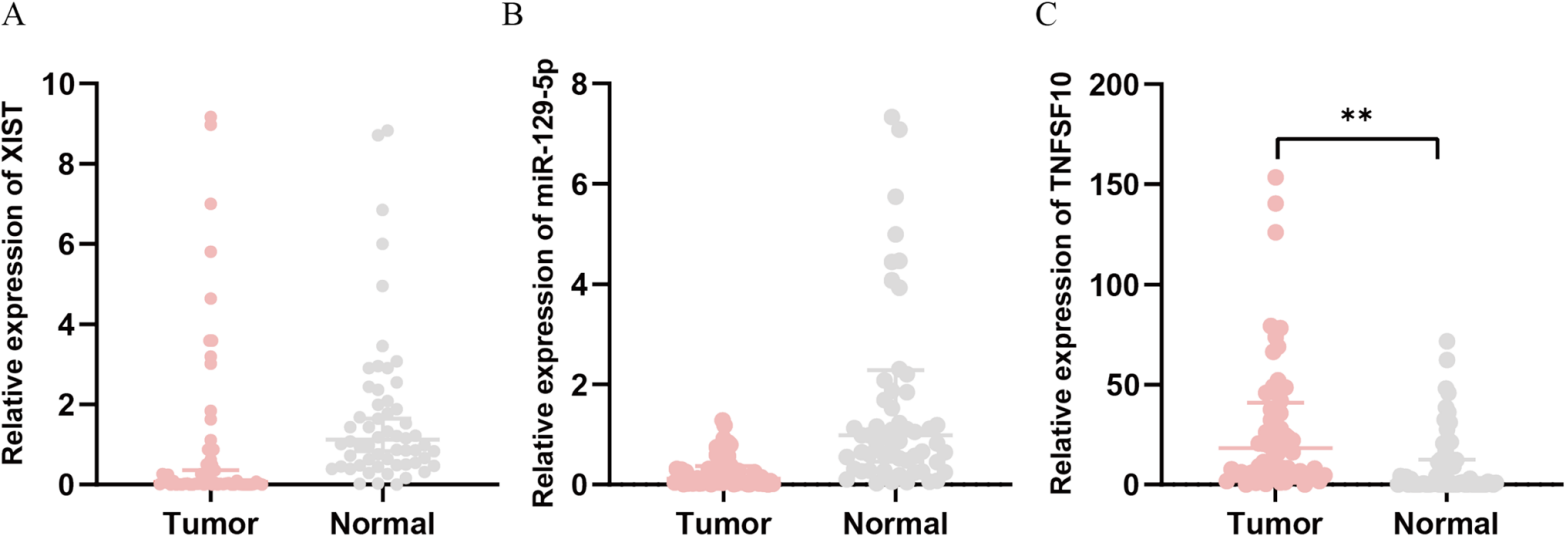
Levels of lncRNA XIST, miR-129-5p and TNFSF10 expression in BC tissues. **A**–**C** qRT-PCR was used to detect the expression levels of lncRNA XIST, miR-129-5p and TNFSF10 in BC tissues and paired adjacent normal tissues, demonstrating that lncRNA XIST and miR-129-5p were downregulated and TNFSF10 was upregulated. ^**^ *P* < 0.01

To further investigate the expression of lncRNA XIST, miR-129-5p and TNFSF10 in BC, we obtained 60 pairs of BC and paired adjacent normal tissues and found that lncRNA XIST and miR-129-5p expression were obviously decreased in BC tissues compared with paired adjacent normal tissues (Fig. 5A, B). Compared to paired adjacent normal tissues, the expression of TNFSF10 was significantly increased in BC tissues (Fig. 5C).

### Correlations between the expression of lncRNA XIST, miR-129-5p and TNFSF10 and clinicopathological features in BC patients

According to the expression levels of lncRNA XIST, miR-129-5p and TNFSF10, all cases were divided into two groups, including: high expression group and low expression group, respectively. The Chi-square test was used to explore the relationship between the expression of lncRNA XIST, miR-129-5p and TNFSF10 with gender, age, T stage and pathological grade of BC patients. As shown in Table 4, lncRNA XIST was significantly correlated with gender. Specifically, the proportion of men with high expression of lncRNA XIST was lower than that of women (*P* = 0.002). A high level of miR-129-5p was significantly correlated with poor pathological grade (*P* = 0.039). However, TNFSF10 expression was not associated with gender, age, T stage and grade.

**Table 4:** Correlations between the expression of lncRNA XIST, miR-129-5p and TNFSF10 and clinicopathological features in BC patients

Clinicopathological Parameters	Case	lncRNA XIST		*P* value	Case	miR-129-5p		*P* value	Case	TNFSF10		*P* value
		Low	High			Low	High			Low	High	
Gender				0.002				0.390				0.774
Male	43	27	16		43	23	20		43	21	22	
Female	17	3	14		17	7	10		17	9	8	
Age(years)				0.100				0.273				0.584
≤ 60	20	7	13		20	8	12		20	9	11	
> 60	40	23	17		40	22	18		40	21	19	
T stage				0.404				0.931				0.471
Ta	24	14	10		24	14	10		24	9	15	
T1	6	4	2		6	3	3		6	3	3	
T2	17	8	9		17	8	9		17	9	8	
T3	6	2	4		6	3	3		6	3	2	
T4	5	1	4		5	2	3		5	4	1	
Grade				0.115				0.039				
Low	13	9	4		13	10	3		13	6	7	0.753
High	45	20	25		45	20	25		45	23	22	

### Diagnostic value of lncRNA XIST, miR-129-5p and TNFSF10 in BC patients

**Fig. 6:**
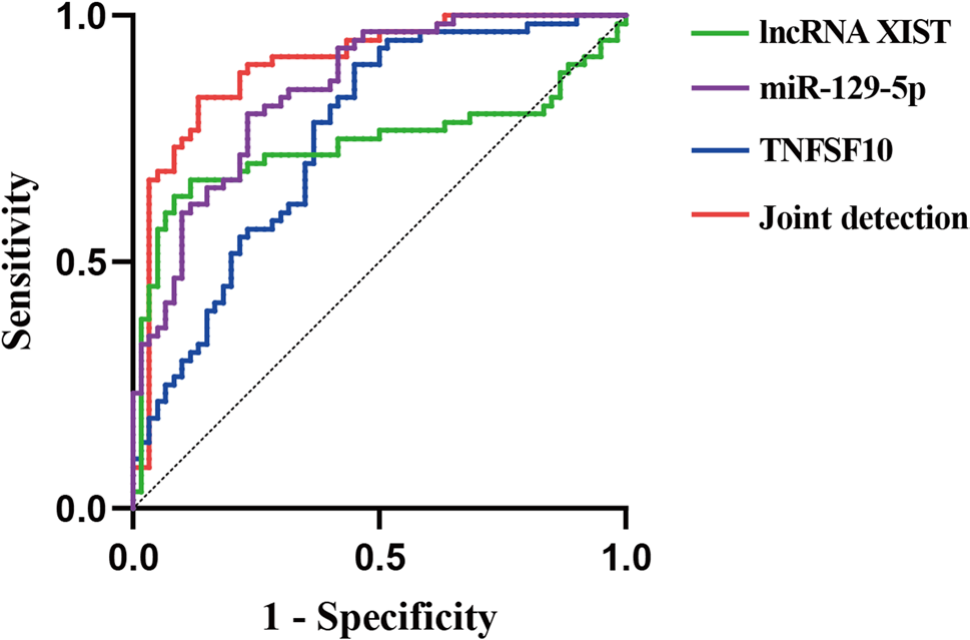
Diagnostic value of the three genes determined by ROC curves

The diagnostic values for lncRNA XIST, miR-129-5p and TNFSF10, and three gene combinations in discriminating between BC and adjacent healthy tissues were determined using ROC curve analysis. As shown in Fig. 6 and Table 5, the AUCs for lncRNA XIST, miR-129-5p and TNFSF10 were 0.739(95% CI 0.641–0.836), 0.850(95% CI 0.784–0.917) and 0.753(95% CI 0.667–0.840), respectively. To assess the cumulative performances of these genes, a binary logistic regression was adopted. The logistic regression model showed that the combination of these three genes could provide the best diagnostic accuracy, with the AUC of 0.900 (95% CI 0.842–0.958) (Fig. 6 and Table 5).

**Table 5:** ROC curve data

Indicators	AUC	95%CI	Sensitivity %	Specificity %	Cut-off
lncRNA XIST	0.739	0.641–0.836	63.3	91.7	0.281
miR-129-5p	0.850	0.784–0.917	80.0	76.7	0.417
TNFSF10	0.753	0.667–0.840	90.0	55.0	1.638
Joint detection	0.900	0.842–0.958	83.3	86.7	—

## Discussion

**Fig. 7:**
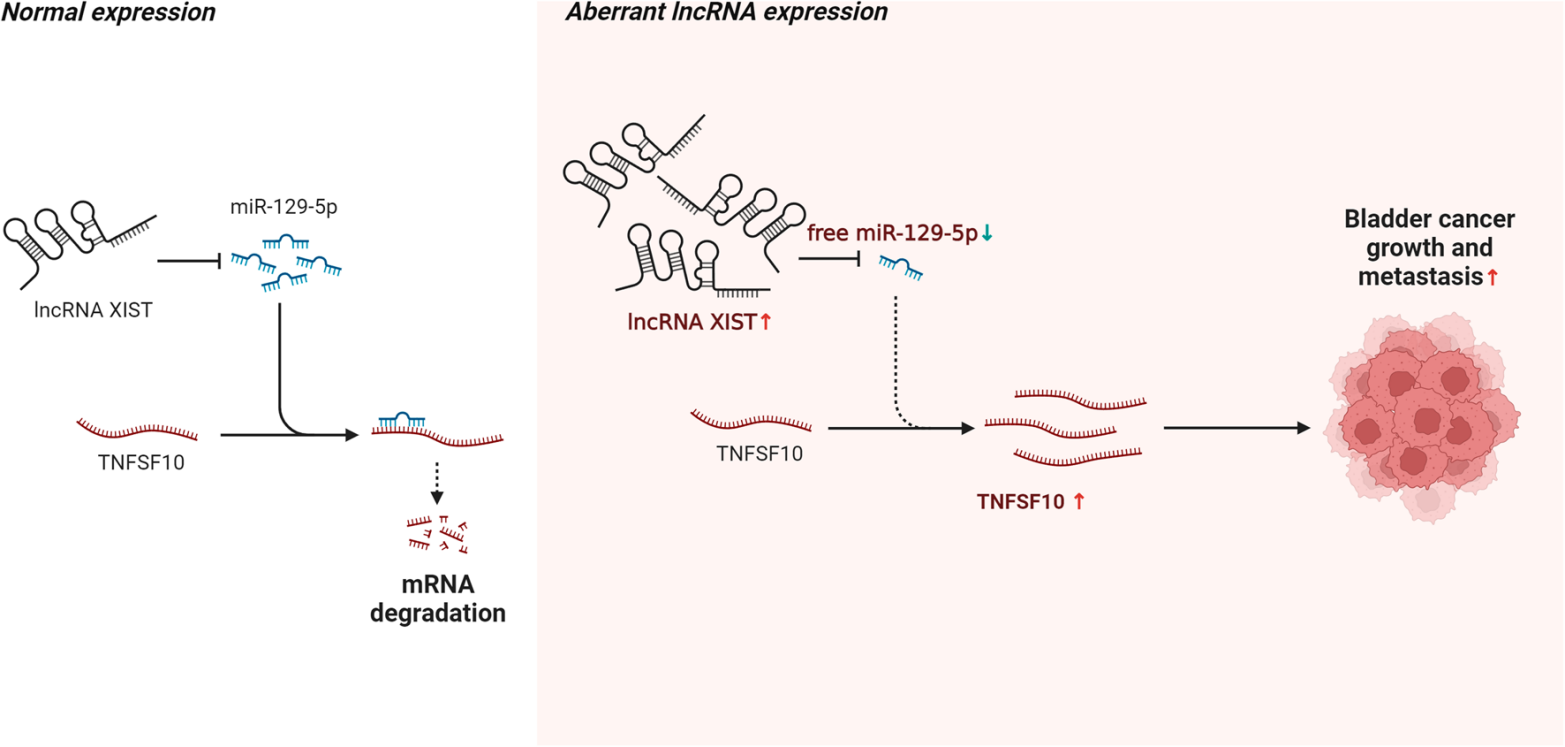
Schematic illustration indicates the mechanism by which lnRNA XIST acts as a tumor promoter in BC through regulating the miR-129-5p/TNFSF10 axis (The schematics was generated using BioRender.com)

The malignancy of the urinary system, known as BC, is considered to be one of the most perilous conditions, with the sixth highest incidence among men [1]. BC is broadly classified into non-muscle invasive bladder cancer (NMIBC) (Ta-T1) and muscle invasive bladder cancer (MIBC) (T2-T4) according to the degree of infiltration, with NMIBC accounting for approximately 80% of new cases, but its recurrence and progression rates remain high [14] and its treatment outcome remains far from satisfactory due to the lack of effective diagnosis and therapeutic approaches. With the development of molecular biology, new molecularly targeted therapeutic strategies are urgently needed to improve the early diagnosis and survival of BC. Hence, the study revealed a novel regulatory mechanism of lncRNA XIST to promote bladder cancer progression through modulating miR-129-5p /TNFSF10 axis (Fig. 7).

Previous studies have pointed out lncRNA XIST was elevated in gastric [15], glioma [16], pancreatic [17], and colorectal cancers [18]. LncRNA XIST was uncovered to be overexpressed in our findings, which could aggravate the malignant behaviors of BC. As proposed by the ceRNA hypothesis, lncRNA can act as a ceRNA to sponge targeted miRNAs and participate in the regulation of biological events [5]. Thus, we speculated that lncRNA XIST might act as a ceRNA in this study. Here, miR-129-5p that bind with lncRNA XIST and TNFSF10 were screened by bioinformatic analysis together with luciferase reporter assays. miRNAs were documented to play a critical role in cancers and so had been emerged as novel molecular markers for tumor diagnosis and prognostic assessment [19]. miR-129-5p was an important molecule involved in the regulation of cell proliferation, migration, apoptosis and signaling pathways [20, 21]. miR-129-5p was identified to decreased in prostate cancer and may function as a tumor suppressor via repression of ETV1 [22]. Besides, another study has demonstrated that miR-129-5p may suppress cell proliferation of osteosarcoma cancer by down-regulating Lnc712 [21]. Similarly, in this study, we found that miR-129-5p was lowly expressed while expression levels of TNFSF10 were highly expressed in BC than in normal uroepithelial cell line. Based on this, lncRNA XIST could sponge miR-129-5p to modulate TNFSF10 expression in bladder cancer through the predicted binding site in terms of the mechanism.

On the basis of the cellular experiments, we found in further histological experiments that lncRNA XIST and miR-129-5p were lowly expressed and TNFSF10 was highly expressed in BC tissues compared to paired adjacent normal tissues. In previous cellular experiments, we have confirmed that lncRNA XIST is highly expressed in BC cells, which is consistent with previous studies [13, 23], but the inconsistent expression in population specimens may be due to three possible reasons. First, the sample size of collected BC patients was small, and there was a large heterogeneity among BC patients. Second, lncRNA XIST might have a relationship with clinical stages of BC. Third, the expression of lncRNA XIST might be influenced by other factors that we have not completely collected. We are interested in further expanding the sample size or constructing animal models of BC for further validation in future studies.

In relation to the total number of human genomes, the availability of functionally annotated lncRNAs and miRNAs in cancer remains limited. Therefore, elucidating novel dysregulated lncRNAs and miRNAs, particularly lncRNA-miRNA interactions that play a crucial role in the initiation, development, and prognosis of tumor, will unveil new therapeutic targets. Ideally, precise targeting strategies should be developed to selectively inhibit lncRNA-miRNA interactions. By targeting lncRNA-miRNA interactions rather than individual non-coding RNAs, the diagnostic efficiency for BC patients can be enhanced while minimizing the risk of off-target effects. The above data helped initially reveal the presence of lncRNA XIST/ miR-129-5p/ TNFSF10 regulatory network in BC. Meanwhile, our research had several limitations: (i) our research was limited to cell lines cultured in vitro; (ii) the number of tissue samples was small, long-term follow-up data were lacking for analysis, and a large number of clinical samples still needed to be studied to validate and evaluate its stability and reliability as a diagnostic marker for BC in combination with clinical prognostic characteristics; (iii) further collection and clarification of factors affecting ncRNA were needed. Additionally, the application of lncRNA XIST, miR-129-5p and TNFSF10 in the clinical treatment of BC also needs in vivo experiments.

## Conclusions

LncRNA XIST was elevated in BC cells, functioning as a tumor promoter that facilitated the malignant behaviors of BC. Moreover, lncRNA XIST competitively sponged miR-129-5p to regulate the expression of TNFSF10. Furthermore, miR-129-5p and TNFSF10 were significantly associated with the risk of BC, and the combination of the three genes was more valuable for the diagnosis of BC. Hence, the lncRNA XIST/miR-129-5p/TNFSF10 axis might be critical for regulating the development and progression of BC which enriched the ceRNA regulatory network and biological mechanism of lncRNA XIST in BC, and also provided a theoretical basis for targeting ncRNA for BC treatment.

## Data Availability

The data used to support the findings of this study are available from the corresponding author upon request.
